# Active identification of vertebral fracture in the FLS model of care

**DOI:** 10.1007/s11657-023-01289-9

**Published:** 2023-06-29

**Authors:** Francisco J. Rubiño, Antonio Naranjo, Amparo Molina, Sonia Fuentes, Fabiola Santana, Ricardo Navarro, Arturo Montesdeoca, Tito Fernández, José A. Lorenzo, Soledad Ojeda

**Affiliations:** 1 Department of Rheumatology, Hospital Universitario de Gran Canaria Dr. Negrín, Reumatología Barranco de La Ballena, 35011 Las Palmas, Spain; 2 University of Las Palmas de Gran Canaria, Las Palmas, Spain; 3 Department of Orthopedics, Spine Unit, Hospital Universitario de Gran Canaria Dr. Negrín, Las Palmas, Spain

**Keywords:** Vertebral fracture, Fracture liaison service, Osteoporosis, Vertebral fracture assessment, Bone densitometry, Treatment adherence

## Abstract

***Summary*:**

The identification of vertebral fracture is a key point in an FLS. We have analyzed the characteristics of 570 patients according to the route of identification (referral by other doctors, emergency registry or through VFA), concluding that promoting referral by other doctors with a training campaign is effective.

**Purpose:**

Vertebral fractures (VF) are associated with increased risk of further VFs. Our objective was to analyze the characteristics of patients with VF seen in a Fracture Liaison Service (FLS).

**Methods:**

An observational study was carried out on patients with VF referred to the outpatient metabolic clinic (OMC) after a training campaign, identified in the emergency registry, and captured by VF assessment with bone densitometry (DXA-VFA) in patients with non-VFs. Patients with traumatic VF or VF > 1 year, infiltrative or neoplastic disease were excluded. The number and severity of VFs (Genant) were analyzed. Treatment initiation in the first 6 months after baseline visit was reviewed.

**Results:**

Overall, 570 patients were included, mean age 73. The most common route for identifying VF was through referral to OMC (303 cases), followed by the emergency registry (198) and DXA-VFA (69). Osteoporosis by DXA was found in 312 (58%) patients and 259 (45%) had ≥ 2 VFs. The rate of grade 3 VFs was highest among patients on the emergency registry. Those identified through OMC had a higher number of VFs, a higher rate of osteoporosis, more risk factors and greater treatment initiation. Patients with VFs detected by DXA-VFA were mostly women with a single VF and had a lower rate of osteoporosis by DXA.

**Conclusions:**

We present the distribution of VFs by the route of identification in an FLS. Promoting referral by other doctors with a training campaign may help in the quality improvement of the FLS-based model of care.

## Introduction

The risk of major osteoporotic fracture (MOF) is two-fold higher after a first MOF, and the imminent risk (in the first 2 years after a fracture) is even higher [1]. If prompt short-term treatment were able to reduce this elevated acute risk, it could be considered clinically beneficial and cost-effective. The risk of subsequent fractures is especially high after a vertebral fracture (VF). In a Canadian cohort that included 67,271 women, recent VF (less than 2 years) was found to be associated with the highest risk of new MOF in the following 2 years (adjusted OR 5.94 for those under 65 years and 2.69 for those over 65), followed by recent humerus and hip fractures [2]. Secondary fracture prevention in VF patients remains suboptimal, and there is a need to close this care gap even within established services. Fracture liaison service (FLS)-based care is associated with more frequent initiation of treatment, reduced mortality and lower subsequent fracture risk [3–5]. In addition, the FLS model is cost-effective compared to standard care [6, 7].

The identification of patients with VF is one of the performance indicators of an FLS, together with early care after the index fracture (< 12 weeks), the percentage of patients with hip fractures, the assessment protocol used, and rates of initiation of and adherence to treatment. Unfortunately, most VFs still go unrecognized or undetected. Regarding secondary fracture prevention, the International Osteoporosis Foundation launched *Capture the Fracture*, a global campaign to break the fragility fracture cycle. Its Best Practice Framework serves as a tool to award ‘*Capture the Fracture* Best Practice Recognition’. Within this framework, the standard related to VF seeks to classify the systems a hospital has put in place to identify VFs among patients seen for other conditions (Table 1) [8]. This is considered important as the prediction of the risk of future fractures is significantly improved by including VF status as well as bone densitometry (DXA).

**Table 1:** Standards for vertebral fracture identification in the Fracture Liaison Service model (Capture the Fracture, Best Practice Recognition) [8]

Level 1	Level 2	Level 3
Patients with clinical vertebral fractures undergo assessment and/or receive treatment for prevention of secondary fractures	Patients with non-vertebral fractures routinely undergo assessment with lateral vertebral morphometry by bone densitometry (or possibly by plain spine radiology) to assess for vertebral fractures	Patients who are reported by the Institution’s Radiologists to have vertebral fractures on plain X-rays, computed tomography & magnetic resonance imaging scans (whether these are serendipitous or not) are identified by the Fracture Liaison Service in order that they undergo assessment for treatment for prevention of secondary fractures

Therefore, one of the objectives when implementing an FLS is to increase the percentage of patients with VFs to between 20 and 40% of all cases. The routes for identifying this type of fracture are through: 1) the emergency registry; 2) referral by other physicians external to the FLS (hospital and primary care) through interprofessional consultation and adequate reporting of the presence of fracture on radiology reports; and 3) systematic performance of lateral spinal radiology and/or vertebral fracture assessment with densitometry (DXA-VFA) in patients with non-vertebral fractures. Considering these routes, the effectiveness of an FLS could be greatly enhanced by the proactive identification of VFs, in particular by radiologists, and the implementation of training programs for other specialties and primary care in the health region.

Our objective was to analyze the characteristics of patients with fragility VFs seen in our FLS as a function of the route of identification. We believe that this type of analysis may help improve the management of FLS, which are currently the standard in secondary fracture prevention [9, 10].

## Methods

We conducted a study of patients with VFs managed through our FLS program. Our FLS opened in 2012, initially serving patients on the emergency registry. From this list, patients are identified using the ICD 10 code and are invited to the program by phone by a nurse specialist. Subsequently, patients seen in our outpatient metabolic clinic with fragility VF were included in the program. Patients referred to this clinic from hospital or primary care are scheduled on the same agenda as patients from the emergency registry for a one-day “high-resolution” consultation managed by a nurse specialist. In addition, since 2018, DXA-VFA has been added to the care pathway for patients with fractures of the humerus or forearm, with the aim of detecting asymptomatic VFs that could influence the choice of treatment and patients with VFs identified in this way are also invited to join the program.

One of the aspects of our FLS is the training program in hospital and in primary care. These are two 45-min sessions with special emphasis on VF, including orthopedic surgeons, rehabilitators, radiologists, geriatricians, and primary care physicians.

### FLS protocol

**Fig. 1:**
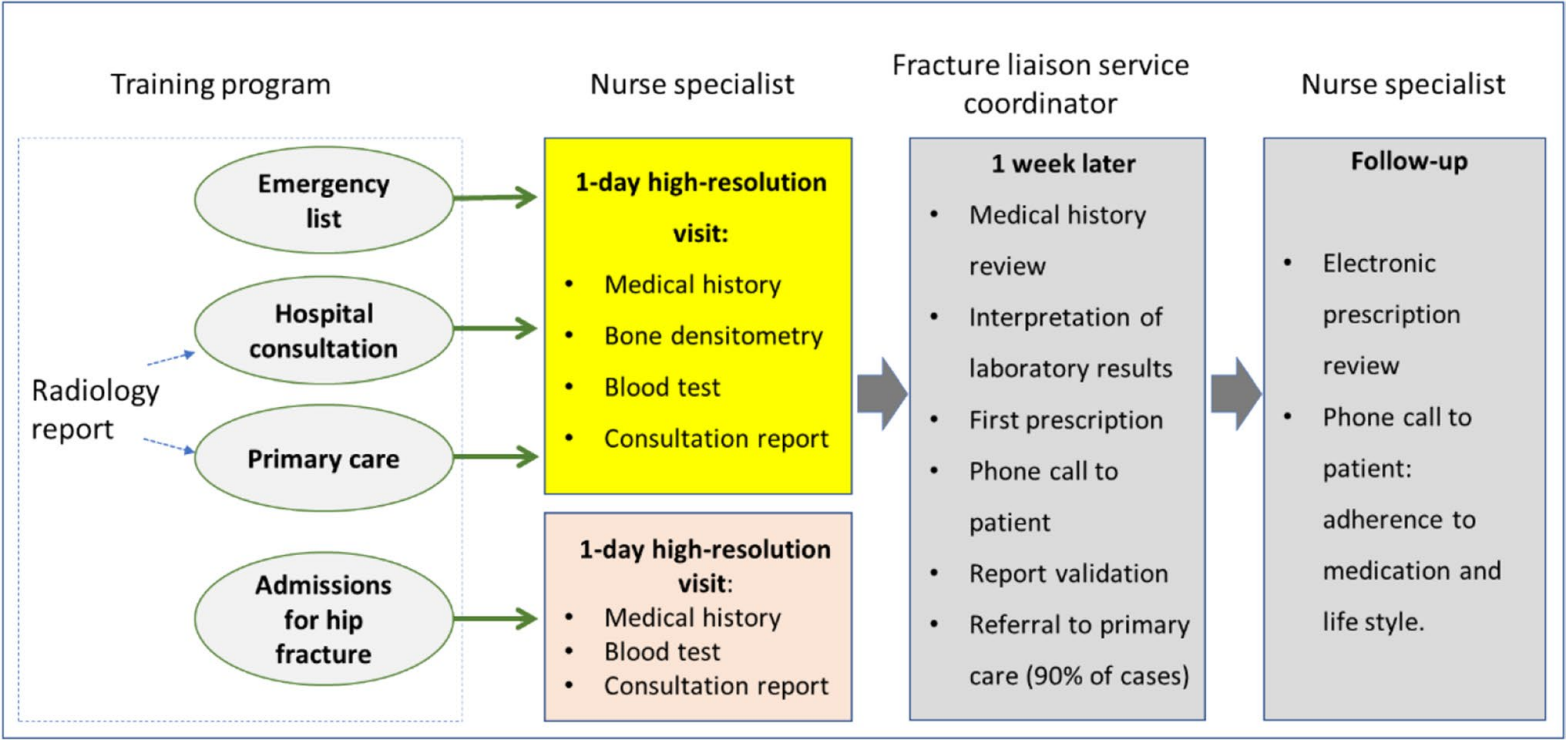
Flow of patients in the Dr. Negrin Fracture Liaison Service (FLS). Patients identified in electronic emergency records with a relevant ICD code (major fractures) are invited to the FLS by phone. Patient referral is occasionally triggered by a radiology report documenting possible vertebral fracture

In the one-day high-resolution consultation, the procedure is as follows (Fig. 1): 1) Medical history focused on bone metabolism, including relevant diagnoses, Fracture Risk Assessment Tool (®FRAX) items as well as history of falls, current and previous treatments and specific factors influencing the choice of medication (e.g., renal function, and dental health); 2) DXA of the lumbar spine and hip, assigning the densitometric diagnosis based on the lowest value obtained at either site; 3) collection of blood sample for basic analysis of bone metabolism (complete blood count, renal function tests, erythrocyte sedimentation rate, and calcium, phosphate, alkaline phosphatase and 25-hydroxyvitamin D levels), adding total protein, calcium, and hormone tests and screening for celiac disease if secondary osteoporosis is suspected; 4) entry of data in a specific FLS database; and 5) provision of patient education on diet, exercise, and fall prevention. At the end, 6) patients receive a written report with provisional recommendations and telling them that a week later they will be contacted by phone by the program coordinator; and 7) the coordinator reviews the patient's complete file (including the results of the blood tests and lateral chest or spine X-rays when available), then contacts the patient by phone to explain the specific treatment indicated and prescribes the initial treatment on the electronic platform of the health region, and finally, the medical report is validated electronically. Note that bisphosphonate, denosumab, teriparatide or romosozumab is recommended per protocol to all patients, and that the flow of patients shown in Fig. 1 reflects the scheme operating since 2020; previously most patients were referred to their primary care physician for the first prescription of treatment. Subsequently, 8) the nurse specialist monitors treatment initiation and lifestyle, including falls, from months 3 to 6. The procurement of medication from a pharmacy is reviewed and taken into account whether a patient is adhering to the treatment prescribed.

Patients with hip fractures identified during admission follow the same protocol as those seen in the high-resolution consultation, except that DXA is not performed (Fig. 1). We performed annual training activities in primary care (for both doctors and nurses), as well as orthopedics, rehabilitation and radiology departments.

### Vertebral fracture assessment

The number and severity of fractures were analyzed in each case using the Genant semiquantitative scale [11]. In all cases, the assessment was carried out by the same observer (AN), assigning grades between 1 and 3: Grade 1 (mild): 20–25% height loss; Grade 2 (moderate): 26–40% height loss; and Grade 3 (severe): > 40% height loss. Regarding DXA-VFA automated result, only grade 2 and 3 fractures were included.

### Statistical analysis

A descriptive statistical analysis was performed. Differences between groups were assessed using the one-way analysis of variance and Wilcoxon tests in the case of continuous variables and the chi-square and Fisher’s exact tests in the case of ordinal or categorical variables. Data were analyzed using IBM SPSS statistics version 27. A p-value less than 0.05 was considered statistically significant.

## Results

Over 11 years, 570 patients with fragility VF have been included, 90 men (15%) and 480 women (84%), with a mean age of 73 years (Table 2). The most common route of identification was through referral by hospital or primary care physicians (*n* = 303 cases) followed by the emergency registry (*n* = 198) and detection by DXA-VFA (*n* = 69). The patients identified by VFA did not report symptoms of VF, and nor did 25 of those referred to our metabolic clinic, with fractures documented in reports by radiologists or detected by rheumatologists. That is, 94 of all the cases (16%) were morphometric VFs. The rest of the cases referred by other doctors and all those on the emergency registry were symptomatic.

**Table 2:** Characteristics of patients with vertebral fracture managed by the Fracture Liaison Service by route of identification. Results are expressed as *n* (%) and mean (SD) unless expressly indicated

	All patients *N* = 570	Emergency registry *N* = 198	Referral to metabolic clinic *N* = 303	Vertebral fracture assessment *N* = 69	*p*
Age, years	73.6 (9.7)	75.5 (9.4)	72.6 (9.9)	72.4 (8.8)	0.003
Sex, women	480 (84.2)	156 (78.7)	261 (86.1)	63 (91.3)	0.020
*Time from sentinel fracture to visit (weeks)*					
Mean	17 (11)	13 (8)	20 (12)	13 (8)	0.02
Median (IQR)	13 (8–20)	12 (8–16)	13 (8–28)	12 (8–16)	
< 12 weeks	214 (37)	83 (42)	101 (33)	30 (43)	0.08
*Vertebral fractures*					
Number of fractured vertebrae					
mean	2.0 (1.6)	1.76 (1.4)	2.35 (1.8)	1.22 (0.5)	
median (IQR)	1 (1–2)	1 (1–2)	2 (1–3)	1 (1–1)	0.000
range	1–12	1–10	1–12	1–4	
Only grade 1 fractures	24 (4.2)	3 (1.5)	20 (6.6)	*	0.007
Two or more fractured vertebrae**	259 (45.7)	78 (39.3)	169 (56.1)	12 (17.4)	0.000
At least one grade 3 fracture***	274 (48.8)	124 (63.9)	141 (47.3)	9 (12.0)	0.000
*Risk factors for fracture*					
Previous fracture	155 (27.2)	51 (25.8)	89 (29.3)	15 (20.0)	0.362
Parental hip fracture	51 (8.9)	20 (10.1)	25 (8.2)	6 (8.0)	0.775
Active smoking	67 (11.7)	21 (10.6)	34 (11.2)	12 (16.0)	0.294
Glucocorticoid use	51 (8.9)	9 (4.5)	40 (13.2)	2 (2.7)	0.001
Rheumatoid arthritis	22 (3.8)	5 (2.5)	17 (5.6)	0	0.045
Secondary osteoporosis	86 (15.0)	37 (18.6)	36 (11.8)	13 (17.3)	0.075
Excessive alcohol intake^†^	21 (3.6)	9 (4.5)	10 (3.3)	2 (2.7)	0.712
Body mass index, kg/m^2^	27.5 (5.0)	28.5 (4.7)	26.2 (5.3)	28.7 (5.1)	0.000
FRAX major osteoporotic fracture	12.7 (8.6)	13.3 (9.6)	13.0 (11.0)	10.8 (7.9)	0.156
FRAX hip fracture	5.6 (6.6)	6.0 (7.9)	5.9 (4.0)	4.3 (6.6)	0.231
Number of falls in the last year					
mean	1.1 (1.0)	1.2 (0.9)	0.9 (1.0)	1.5 (1.0)	
median (IQR)	1.0 (1–1)	1.0 (1–1)	1.0 (0–1)	1.0 (1–1)	0.000
range	0–4	0–4	0–4	1–5	
*Bone densitometry #*					
Normal	46 (8.6)	18 (10.0)	18 (6.3)	10 (13.3)	0.000
Osteopenia	175 (32.8)	69 (38.5)	75 (26.3)	31 (41.3)	
Osteoporosis	312 (58.5)	92 (51.3)	192 (67.3)	28 (37.3)	
Lumbar spine T-score	-2.29 (2.0)	-2.0 (1.7)	-2.5 (2.3)	-1.4 (1.5)	0.000
Femoral hip T-score	-1.91 (1.0)	-1.8 (1.2)	-2.0 (1.0)	-1.5 (0.9)	0.003
*Osteoporosis medication*^&^					
Previous treatment	124 (21.8)	35 (17.6)	80 (28.0)	9 (12.0)	0.012
Treatment initiation within 6 months of the visit	474 (83.1)	161 (81.3)	265 (87.4)	48 (69.5)	0.001

^*^Only grade 2 and 3 fractures were included in vertebral fracture assessment with densitometry

**Available in 567 patients

***Available in 561 patients

^†^more than 3 units/day

^#^Available in 533 patients

^&^Bisphosphonate, denosumab, a selective estrogen receptor modulator, or teriparatide

**Fig. 2:**
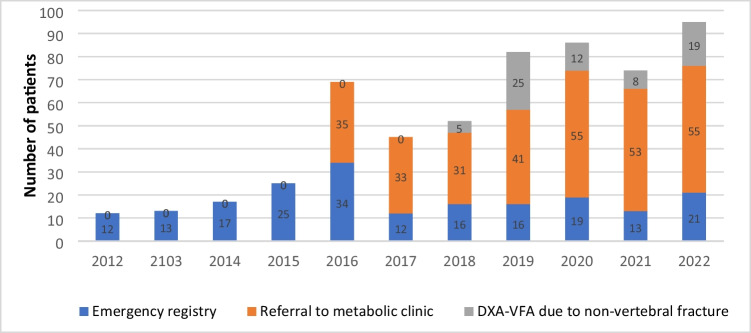
Identification of vertebral fractures in 2012–2022. DXA-VFA: vertebral fracture assessment with densitometry

Figure 2 shows changes in the route of patient identification over the years. In 2022, the distribution of fractures was as follows: 58% were referred to our clinic (25% from hospital specialties and 33% from primary care), while 22% were identified through the emergency registry and 20% through DXA-VFA in patients with other fractures. Overall, 21 of 95 (22%) VFs identified that year (2022) were morphometric; of these, 19 were silent VFs detected by DXA-VFA and 2 were incidental findings in a radiology report.

Table 2 summarizes the characteristics of each group. The time from the sentinel fracture to the baseline visit was an average of 17 weeks, being longer in patients referred to the clinic than in those identified on the emergency registry (20 vs 13 weeks; *p* = 0.02). Nonetheless, if the date of the referral to the clinic is taken into account instead of the date of the fracture, the average waiting time was 4 weeks, with 84% of patients identified through referral being seen before 12 weeks.

Among cases identified on the emergency registry, patients were 3 years older and more likely to have grade 3 fractures. On the other hand, patients identified through referral to the clinic were characterized by having more fractures, a higher rate of osteoporosis by DXA, and a history of fractures, as well as rheumatoid arthritis and glucocorticoid use. Further, patients in this group had higher treatment initiation in the 3–6 months after the fracture. The 69 cases detected by DXA-VFA corresponded to patients with humerus fractures (30 out of 163 analyzed), pelvic ramus fracture (1 out of 4 analyzed), or forearm fractures (38 out of 193 analyzed). In this group, patients were more likely to be women with a single fracture, frequent fallers, and have lower rates of osteoporosis by DXA and lower treatment initiation.

In a multiple regression analysis, the factors associated with grade 3 vertebral facture were identification through the emergency registry or referral to the metabolic clinic (OR 2.72; 95% CI 2.00–3.68), female sex (OR 1.72; 95% CI 1.01–2.94), and a lower femoral neck T-score (OR 1.24; 95% CI 1.05–1.47). The factors associated with two or more VFs were a lower femoral neck T-score (OR 1.28; 95% CI 1.08–1.52) and older age (OR 1.02; 95% CI 1.00–1.04).

Treatment recommendations were as follows: bisphosphonates 53.3%, denosumab 41.2% and anabolics 5.4%. The recommendation of anabolic was significantly higher (*p* = 0.01) in the outpatient group (6.9%) vs the emergency group (3.5%) and the VFA group (0%). No appreciable differences were observed in antiresorptive by groups.

Treatment initiation was higher for denosumab than for teriparatide or bisphosphonates (92%, 91% and 77%, respectively; *p* = 0.09). The VFA group had the lowest treatment initiation of bisphosphonates (63%; *p* = 0.07 compared to other groups).

Regarding treatment initiation, we have observed a change over the years. In 2021 and 2022, after implementing prescribing from the hospital by the FLS coordinator for all patients, we found an overall treatment initiation rate in the first 6 months of 93%, compared to 79% in the earlier period (2012–2020).

### Sex differences

Sex differences were observed in several variables. A higher percentage of male patients were identified on the emergency registry (46% vs 32% in women; *p* = 0.020). Men were more likely to have grade 3 VFs (61% vs 45% in women; *p* = 0.008). Regarding the risk factors, excessive alcohol consumption was more common (14% vs 1%; *p* = 0.000) and secondary osteoporosis was less common in men than women (7% vs 16%; *p* = 0.035). Lastly, men were less likely to be found to have osteoporosis by DXA criteria (42% vs 57% in women; *p* = 0.001).

## Discussion

The results of this study show that promoting early referral for VF from outpatient hospital and primary care is a good strategy in the FLS model. These are patients with a very high risk of refracture, as shown by the fact that more than half were found to have two or more VFs and two-thirds were diagnosed with osteoporosis by DXA criteria.

Our FLS is multidisciplinary with three distinctive characteristics: 1) a very prominent role of nurses, 2) a one-day high-resolution baseline visit, and 3) a close collaboration with primary care centers. It opened in 2012 and we have been improving the model, especially in capturing VFs by different routes [12, 13].

The recommendations of the *Capture the fracture* program [8] indicate that patients should be seen in an FLS no more than 16 weeks after the fracture occurred, and ideally within 8 weeks. We found an average delay of 17 weeks, with 37% of cases being seen within 12 weeks, similar to in other FLS [14]. On the other hand, our hospital´s quality assurance unit has recommended modifying the indicator for patients referred by doctors external to the FLS: in such cases, they suggested using the date of referral instead of the date of fracture. Applying this criterion to patients identified through referral to the FLS metabolic clinic, the percentage of patients seen in the first 12 weeks increased to 66%.

Identification of fragility VF is of the utmost importance to prevent new fractures, since this is the type of fracture that is most strongly associated with densitometric diagnosis of osteoporosis and most increases the risk of new fractures [2, 15]. Moreover, there are several effective treatments for the secondary prevention of fractures. Analysis of the impact of the fractures documented in radiology reports or detected by VFA on the practice of an FLS is rarely reported in the literature. The results of the present study indicate that the patients identified by DXA-VFA have fewer VFs and those they have are less severe. By contrast, patients identified through referral to the clinic have more fractures and are more likely to be found to have osteoporosis by DXA. This group of patients referred by other doctors showed the highest rate of treatment initiation rate (close to 90%).

In our experience, about half of VFs are actively identified through the emergency registry or by DXA-VFA. For years, we have carried out repeated training programs in primary care and the radiology department, and this has been associated with an increase in the referral of fractures. Notably, in the last year 58% of the cases analyzed were referred by other physicians, and this is likely to be in part due to the impact of the training program. This idea is supported by the trend seen in Fig. 2. We have asked radiologists to send us a list of fractures monthly, but they have not met this target and the information was not easy to obtain electronically. What they have started to do, after several teaching sessions, is document the presence of fractures and note that the corresponding patients are candidates for a bone metabolic study to prevent new fractures in their reports.

Compared to the study by Olmos-Montes et al. [14] on the analysis of FLS indicators carried out in Spain, we found a lower percentage of VFs out of all fractures identified. In our FLS, we do not perform lateral spinal X-rays routinely. We do, however, review lateral chest or spine X-rays when available, as well as perform DXA-VFA in patients with fractures of the humerus, forearm, or pelvis. When excluding the morphometric fractures detected in patients with hip fractures in the study by Olmos-Montes et al. [14], the percentage of morphometric fractures (out of all the VFs they detected) decreases from 43 to 21%, which is close to the rate detected in our FLS (20%) through DXA-VFA in 2022 in our FLS.

A VFA-identified VF is associated with an increased risk of incident MOF [16]. Therefore, DXA-VFA is recommended to detect subclinical VFs, which may modify patients’ risk category and treatment (its initiation, type and/or duration), depending on age and local criteria for intervention [17]. In addition, DXA-VFA provides a baseline assessment, based on which later incident VFs can be distinguished from prevalent fractures, critical for optimal treatment monitoring. The role of DXA-VFA in an FLS was analyzed in a large cohort and VFs were identified in 15% of patients [18]. The risk of previously undiagnosed VF was higher for hip, humerus, pelvis, forearm, and other fractures, in that order. The authors concluded that DXA-VFA is useful for risk stratification and monitoring of patients, recommending VFA in FLS patients over 70 years of age with low bone density.

We have previously described an overall treatment initiation rate in hip fracture of 84% in the first 6 months in patients referred to our clinic [19], similar to the figures for VF. Recently, however, since the FLS coordinator took over the task of prescribing the initial treatment, we have observed that the overall treatment initiation rate in patients with VF has risen to 90%.

Our advice to other FLS after 11 years of experience is that all the identification methods are necessary to achieve approximately 30% VF of all fractures identified in an FLS.

Our study has some limitations, such as not systematically performing lateral spinal X-rays in non-VF patients. However, it has been replaced by DXA-VFA. We believe that the results are generalizable due to the sample size and the variety of patients included.

In conclusion, we present the distribution of VFs by the route of identification in an FLS and the associated clinical characteristics. In our case, apart from detection by DXA-VFA, we believe that the training campaign carried out in our health region, including radiologists, has allowed us to achieve acceptable indicators without the need to perform systematic lateral x-rays of the spine, which would break with the high-resolution (single visit) approach of our FLS. Our results can help managing and improving VF identification in the FLS-based model of care.
